# Extravascular implantable cardioverter and leadless pacemaker interactions

**DOI:** 10.1093/europace/euaf255

**Published:** 2025-10-16

**Authors:** Alfonso Aranda Hernandez, G Stuart Mendenhall

**Affiliations:** Medtronic Research and Technology, 8200 Coral Sea Street NE, Mounds View, MN 55112, USA; Scripps Memorial Hospital—La Jolla, La Jolla, CA, USA

**Keywords:** Extravascular ICD, Leadless pacemaker, VF detection

## Abstract

**Aims:**

Traditional cardiac pacing and defibrillation devices rely on leads connected to a subcutaneous pulse generator, which can result in complications such as vascular damage, infection, or lead failure. Advances in technology have led to the development of leadless pacemakers, which combine the battery, circuitry, and electrodes into a single self-contained unit, and extravascular implantable cardioverter-defibrillators (ICDs), which position electrodes outside the vasculature. These innovations offer promising alternatives for patients requiring both defibrillation and pacing, particularly those unable or unwilling to accommodate traditional leads. However, the interactions between extravascular ICDs and leadless pacemakers remain largely unexplored and currently lack regulatory approval for combined use. This study evaluates the interactions between leadless pacemakers and extravascular ICDs to assess their simultaneous operation.

**Methods and results:**

*In-silico* simulations, saline-tank experiments, and Monte Carlo simulations were conducted to evaluate device interactions, focusing on ventricular fibrillation (VF) detection during pacing conditions. Ventricular fibrillation detection was unaffected by pacing pulse widths ≤0.24 ms, with a pacing pulse-to-VF amplitude ratio of ≤2 considered safe. Wider pulse widths or higher outputs progressively increased the risk of VF undersensing. Experiments confirmed that pacing pulses ≤3 V and ≤0.24 ms minimally impacted VF detection. Proximity of device affected pacing pulse amplitude sensed by the ICD, but pacemaker orientation did not. Monte Carlo simulations indicated a 0–4% probability of undesired interactions under clinically relevant conditions.

**Conclusion:**

Extravascular ICDs and leadless pacemakers may safely coexist, with a low observed risk of VF undersensing in our study. Further clinical studies are needed to confirm these findings.

## Graphical Abstract

**Graphical Abstract:**
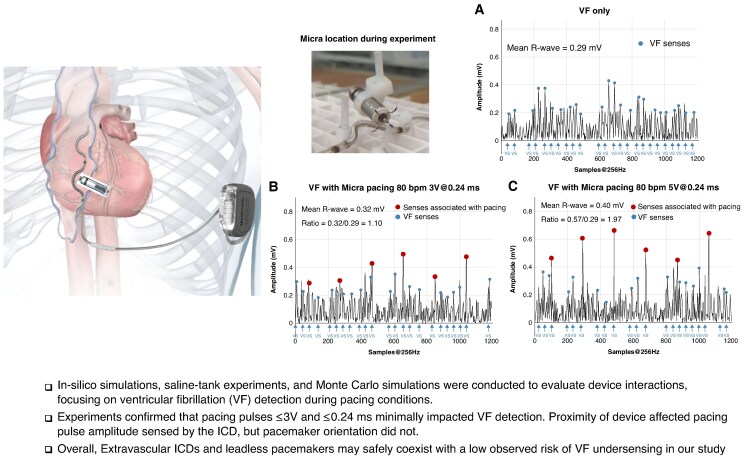

## Introduction

Cardiac implantable electronic devices that deliver therapy have historically been divided into pacemakers, or ‘low energy’ devices, which provide stimulus to make the heart contract in the order of several volts, and implantable cardioverter-defibrillators, or ‘high energy’ devices, which are able to give an electric field that captures large amounts of myocardium through application of several hundred volts. While pacemaker technology finds its origin in the 1950s, defibrillators began to be reduced in size for implantation in the 1980s, and became significantly more common in the late 1990s. Since the late 1990s, all implantable cardioverter-defibrillators (ICDs) have integrated a pacing circuitry. There was a brief period in the 1980s and 1990s, when ICDs and pacemakers were often separate devices, and care had to be used to prevent interaction with inappropriate oversensing of pacemaker spikes or undersensing of ventricular fibrillation.

Leads with electrodes for both pacing and defibrillation typically travel through the venous circulation from the device ‘can’ which contains the battery and circuitry. However, over time leads can adhere to vascular walls, impair circulation, become infected, or perform poorly and therefore require removal or abandonment. With miniaturization of technology, new ‘leadless’ devices that contain the entire battery, circuitry, and electrodes in a single housing have emerged for pacing (2016^1^). Also, extravascular ICDs with electrodes outside the heart in the mediastinum are now commercially available (2014^2^). Unlike transvenous ICDs, extravascular ICDs lack chronic pacing capabilities due to the significant energy demands required to achieve cardiac capture from leads positioned outside the heart. The increased distance between the leads and the myocardium necessitates much higher energy output for pacing, which would rapidly drain the device’s battery and compromise its longevity. Over time, extravascular ICD patients might develop the need for pacing, while those receiving a leadless pacemaker may require protection from SCD, highlighting the potential clinical need for combining these two technologies in selected cases. This highlights a potential problem of device–device interference, in which having separate systems for pacing and defibrillation may lead to inappropriate sensing behaviour. In particular, pacing pulses from a leadless pacemaker can be misinterpreted by the extravascular ICD, resulting in oversensing or undersensing of cardiac signals. Similar challenges have been reported in the broader context of cardiac implantable electronic devices, where electromagnetic fields or device-related artefacts can induce inappropriate sensing. For example, pacemaker and ICD oversensing has been described in patients moving near an MRI scanner bore,^3^ and methods for risk analysis in workers exposed to electromagnetic fields while carrying active implantable medical devices have also been published.^4^ These prior observations underscore the importance of systematically investigating the potential for interference when combining pacemakers with ICDs.

In this paper, we study the mechanism of oversensing and undersensing by a substernal extravascular implantable defibrillator (Aurora EV-ICD, Medtronic, USA)^5^ caused by the pacing pulses from a concomitant leadless pacemaker (Micra, Medtronic, USA).^1^ We describe *in-silico* and saline-tank simulation experiments with real and simulated waveforms to evaluate device performance. We apply Monte Carlo simulations with a distribution of inter-device distances using experimental data and enumerate any conditions of unwanted interference due to over- or undersensing.

## Methods

### Cardiac devices

#### Aurora EV-ICD™ system

**Figure 1:**
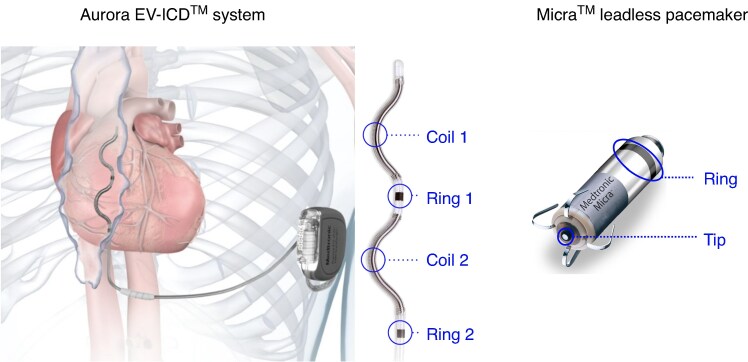
Illustration of Aurora EV-ICD and Micra systems.

The Aurora EV-ICD™ (Medtronic, Minneapolis, MN, USA) system (*Figure 1*) is designed to treat life-threatening heart rhythms, such as sudden cardiac arrest, by delivering defibrillation and antitachycardia pacing (ATP) therapies. Unlike traditional transvenous ICDs, the Aurora EV-ICD system places the lead outside the heart and veins, specifically beneath the sternum, while the defibrillator is implanted below the left armpit. The EV-ICD has an epsilon-shaped lead with two defibrillation coils and two sensing/pacing electrodes (*Figure 1*).

#### Micra™ leadless pacemaker

The Micra™ (Medtronic, Minneapolis, MN, USA) transcatheter pacing system (*Figure 1*) is a leadless pacemaker. Unlike traditional pacemakers that require leads and a surgical ‘pocket’ under the skin, the Micra is implanted directly into the right ventricle of the heart via a minimally invasive procedure through a vein in the leg. This leadless design eliminates potential complications related to leads and reduces recovery time. Throughout the remainder of the manuscript, we will refer to Aurora EV-ICD as ‘ICD’ and to Micra as ‘PPG’ (pacemaker pulse generator).

#### Micra™ and Aurora EV-ICD™ interactions and labelling

In this study, we investigated the interactions between the Micra VR leadless pacemaker and the Aurora EV-ICD system, including scenarios outside current labelling.

Per the Micra approved labelling (for Micra VR/AV/VR2/AV2 currently available models), concomitant use with another device providing active cardiac therapy is contraindicated if the other device may interfere with Micra sensing. Because the EV-ICD device can deliver ATP, post-shock, and pause-prevention pacing, interference risk is not currently defined in the approved labelling. To stay clear of this contraindication, conclusive evidence or justification would presumably be required that the other implanted device could not interfere with Micra sensing. Therefore, the concomitant use of EV-ICD and Micra IPGs is currently off-label. The Micra labelling also identifies a contraindication if another implanted device would interfere with the implant of the Micra device in the judgment of the implanting physician.

Further, per the Aurora EV-ICD approved labelling, concomitant use is contraindicated with unipolar pacemakers, devices delivering dual/triple-chamber pacing, and devices delivering antitachyarrhythmia therapies. The labelling also states that EV-ICD is contraindicated if symptomatic bradycardia exists. The EV-ICD labelling includes a warning regarding concomitant use with a single-chamber bipolar pacemaker. The warning emphasizes the need to verify that the concomitant device maintains capture and correctly senses all intrinsic ventricular rhythms (NSR and ventricular tachyarrhythmias). The labelling warns that inadequate verification could result in inappropriate tachyarrhythmia detection and therapy or undersensing of VF. Our experiments intentionally included off-label configurations to assess feasibility and interaction mechanisms, and these findings do not constitute labelling or clinical recommendations.

### *In-silico* studies

For *in-silico* simulations of interactions between the PPG and the ICD, we used a dataset of 601 de-identified, device-recorded, and verified episodes of true polymorphic ventricular tachycardia and ventricular fibrillation. Of these episodes, two were spontaneous episodes while the remaining were induced during defibrillation testing. The dataset consists of eight-bit electrogram (EGM) recordings obtained from 120 patients enrolled in the EV-ICD Pivotal study.^5^ Recordings are from bipolar vectors using lead Ring1 and Ring2 electrodes and the device ‘can’ electrode, with most recordings from the Ring1-Ring2 configuration. The digitized recordings were extracted from the device transmission file containing the recording. The sampling rate of the recording was 256 Hz. As recording starts after detection of arrhythmia and the intervention typically takes place rapidly, the recorded episodes typically lasted 6–10 s and thus were of insufficient length to simulate an ongoing clinical arrhythmia. In order to simulate a longer period of arrhythmia for testing, the recording episode was looped (electronically stitched) to form a repeated VT/VF signal of 60 s duration. To mitigate discontinuity artefacts from the looping process, connection points near isoelectric values were selected to minimize amplitude spikes at junctions. The polynomial spline method was used to ensure a smooth connection.

**Figure 2:**
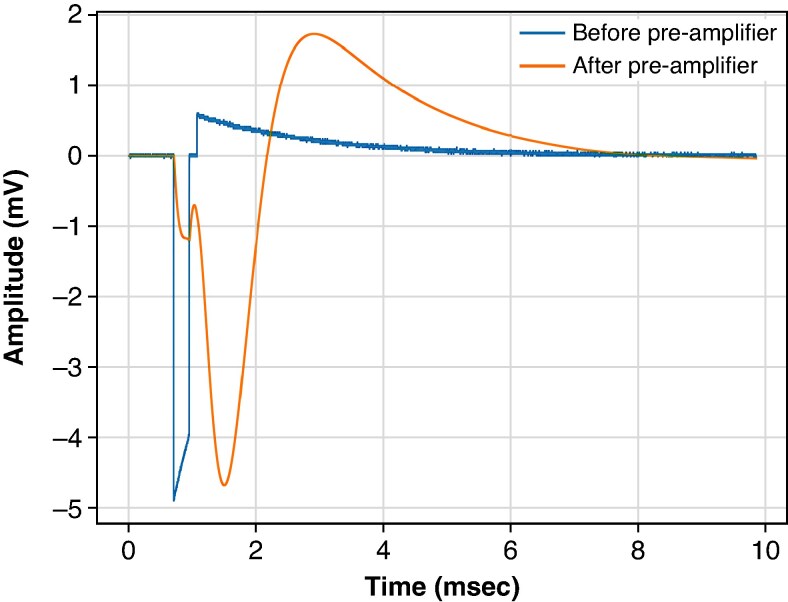
Example of the pre-amplifier effect in pacing pulses. The simulated raw pacing pulse has an amplitude of 5 mV and pulse width of 0.24 ms. The initial spike represents the pacing pulse in the model’s input (simulating the pacing pulse picked up at the sensing electrodes Ring1 to Ring2, while the later smoothed potential represents the pacing pulse output by the model (simulating the pacing pulse post-EGM pre-amplifier digitally at 1 MHz).

Leadless pacemaker pulses were superimposed on the ICD recordings to simulate asynchronous pacing during the simulated VT/VF episode. Sensing was performed using a MATLAB (Mathworks, Natick, MA, USA) software development version of the sensing algorithm in the substernal ICD obtained from the manufacturer. As the pacemaker pulses would be affected by the local environment and the pre-amplifier, the output reference spike/raw pacing pulse was passed through a model of the ICD pre-amplifier. This typically resulted in a reduction in slew rate and a slight rebound effect, as shown in *Figure 2*.

**Figure 3:**
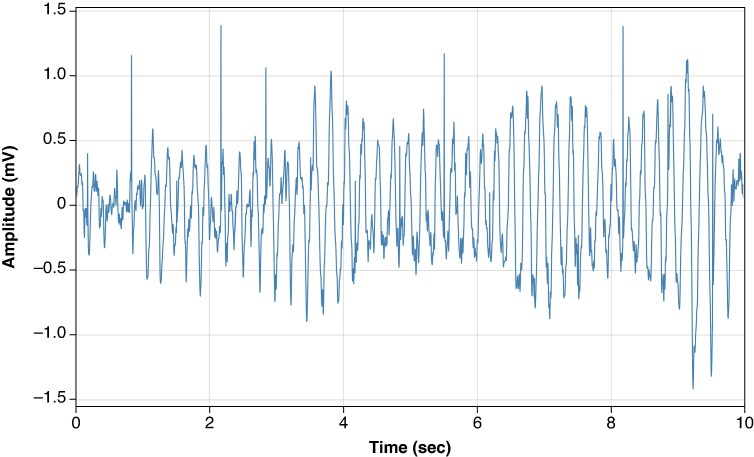
Example of VF recording with pacing spikes of 2.5 mV and 1 ms pulse width.

For all of the VT/VF tracings, asynchronous pacing spikes were added at 60, 90, 150, and 180 bpm to simulate pacing at different physiologic rates (60, 90 bpm) or simulate abnormal conditions (150, 180 bpm). To minimize artefacts in the transition between the pacing pulse extremes and VF, a spline interpolation method was applied. Secondly, the pulse spike amplitude was varied at each rate, expressed as a ratio between the pulse spike and mean VT/VF amplitude. Typical values of the pacing spike/VF amplitude ratio ranged from 0 to 8, consistent with clinical measurement and expectations, which typically have VF sensed amplitude from 0.15 to 1.0 mV and measured pacemaker spike signal of 1–2 mV. A typical recording is shown in *Figure 3*.

### Saline tank studies

**Figure 4:**
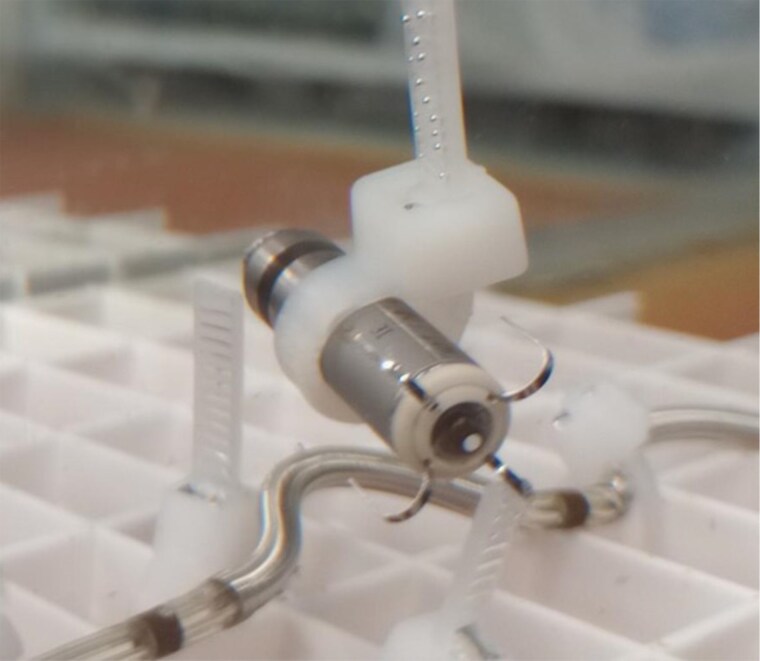
Example of PPG and ICD lead location during saline tank experiments.

**Figure 5:**
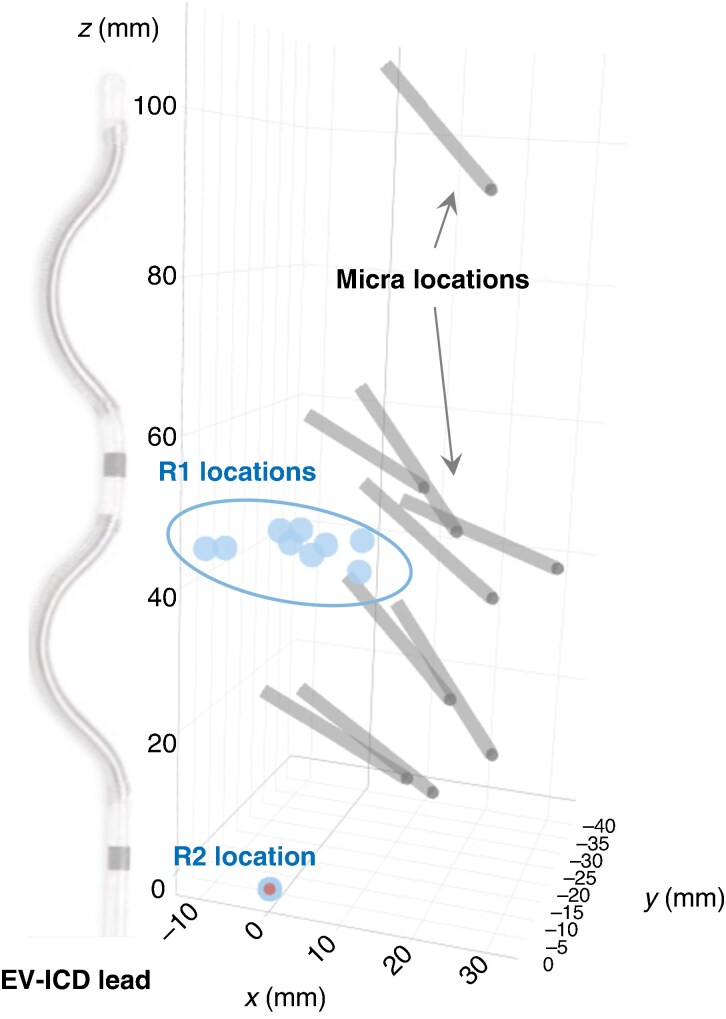
ICD lead and PPG locations for nine patients in the dataset based on anatomy and imaging data. The Ring2 electrode, used as a reference for all patients, is depicted as a point with a darker centre. Other points, contained by a circle, indicate the Ring1 electrode locations for each patient. The grey tubes represent the PPG devices, with their tips marked by black dots.

**Figure 6:**
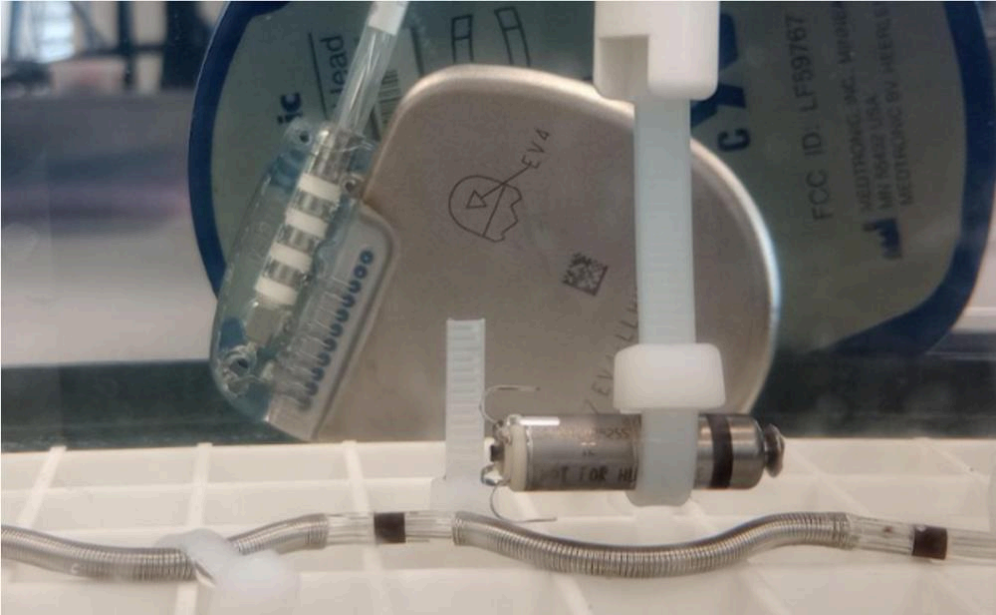
Example of PPG’s closest location experiment in the saline tank. The distance from PPG’s tip to the Ring1 electrode is 16 mm, whereas the distance from PPG’s tail to the Ring2 electrode is 21 mm.

The tank studies experimental setup consisted of a tank containing a saline solution with resistivity of 375 Ω·cm to simulate the typical resistance seen in blood and cardiac tissue. The resistivity of the saline solution was measured using a YSI 3200 Conductivity Instrument (Yellow Springs Instruments, Yellow Springs, OH, USA). The PPG and the ICD devices were located in relative anatomic positions (*Figure 4*) inside the tank using imaging data from nine patients of the EV-ICD Pilot study^6^ and leadless pacemaker implant locations (*Figure 5*). Since our focus was on sensing vectors, only the relative distance between the ICD lead and the PPG was replicated in the tank. Pacing was programmed to amplitudes of 1, 1.5, 3, and 5 V and pulse widths of 0.24, 0.40, and 1.0 ms. The sensed EGM signal (obtained by filtering and rectifying the EGM) was recorded by the ICD for all 12 combinations of pacing amplitude and pulse width and each of the nine relative device positions. Additional data were obtained with the PPG located in a very close location to the ICD lead (*Figure 6*).

**Figure 7:**
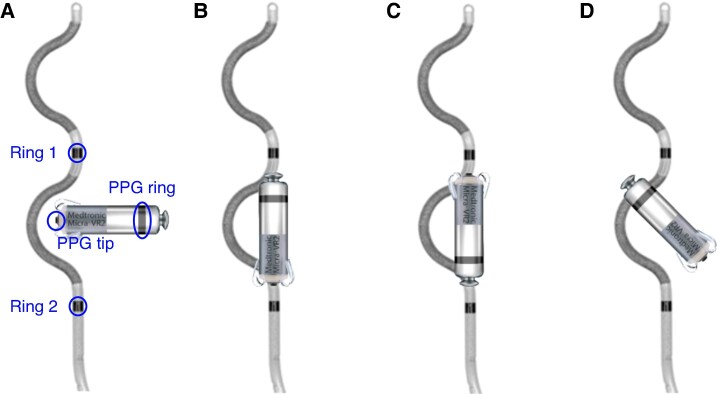
Examples of PPG locations tested during experiments to assess the effect of rotation on pacing.

Three types of saline tank experiments were conducted. In the first experiment, PPG pacing pulses were delivered in the absence of VF, and pacing pulse amplitudes were recorded by the ICD. This experiment aimed to understand the amplitude of the recorded pacing pulses for the different PPG locations. In the second experiment, pacing pulses were delivered asynchronously in the presence of simulated low-amplitude VF to evaluate the interference of the pacing pulses in VF detection. Lastly, a set of experiments was conducted to study the effect of PPG orientation on pacing pulse amplitudes sensed by the ICD. In these experiments, the PPG was in close proximity to the ICD lead, forming angles of 0, 45, 90, and 180 degrees (*Figure 7*).

All saline tank experiments were conducted using Ring1-Ring2 as the primary sensing vector. However, in clinical practice, Ring-Can configurations may be selected when low R-wave amplitudes (<1 mV) are observed at the Ring1-Ring2 vector, in order to reduce the risk of inappropriate therapy from oversensing when the sensing algorithm approaches its sensitivity threshold. To evaluate how pacing spike amplitudes sensed on Ring1-Ring2 translate to the Ring-Can configurations, a comparative analysis of sensed amplitudes across vectors was performed. In this case, measurements were obtained for six different Micra and EV-ICD locations. For each location, pacing was programmed to amplitudes of 1.5, 3.0, and 5.0 V and pulse widths of 0.24, 0.4, and 1.0 ms. In this case, the amplitudes sensed by the EV-ICD were recorded in three sensing vectors: Ring1-Ring2, Ring1-Can, and Ring2-Can. The distribution of sensing amplitudes was summarized using descriptive statistics (median and interquartile range). To assess statistical differences between the sensing configurations, pairwise comparisons were performed using the Wilcoxon signed-rank test. This non-parametric test was chosen due to the potential non-normality of the data. Statistical significance was defined as *P* < 0.05.

### Monte Carlo simulations

Having only nine patients with real-world device location combinations limits the extrapolation of the results to the general population. In this sample, distances between the PPG and ICD electrodes are all >35 mm. The probability of having patients with smaller separations, as well as how VF undersensing relates to pacing output, is unknown. To address this limitation, a Monte Carlo simulation was performed to estimate the probability of patients with expected PPG to ICD electrode distances below 35 mm. The distances from the ICD lead Ring2 electrode to the leadless PPG tip (D1), and from the ICD lead Ring1 electrode to the PPG ring (D2), obtained from the nine concomitant patients, were used as input in the Monte Carlo simulations. The mean and covariance vector of the distances (D1, D2) was then used to create a multivariate normal distribution, from which 50 000 samples were generated.

### Leadless pacemaker use conditions

The risk of VF undersensing is influenced not only by the distance between the PPG and ICD leads but also by the PPG programmed pacing outputs. Therefore, understanding leadless pacemaker programming in clinical practice is crucial. To investigate this, a database of de-identified real-world patient device implant records and remote monitoring transmissions [Medtronic Data Warehouse and Analytics Services (DWAS)] was utilized. From this database, information from ∼24 000 PPGs on ventricular pacing thresholds for both VR (ventricular pacing only) and AV (atrial sensing—ventricular pacing) devices was extracted. Thresholds obtained during the PPG acute implant phase (112 days by default) were excluded as the pacing amplitude is fixed at higher values during this period. There are several potential strategies to manage the high pacing output associated with the acute phase. One approach could be to reduce the default duration from 112 days to a shorter period, and simultaneously lower the PPG sensitivity thresholds to ensure reliable VF detection and prevent PPG pacing during VF. This would eliminate the risk of VF undersensing caused by higher amplitude PPG pacing during a VF episode. Another strategy might involve shortening the 112-day default period while keeping the higher PPG pacing output during this time, but raising the ICD sensitivity threshold. However, this approach carries a higher risk of undersensing of VF.

## Results

### *In-silico* studies

**Figure 8:**
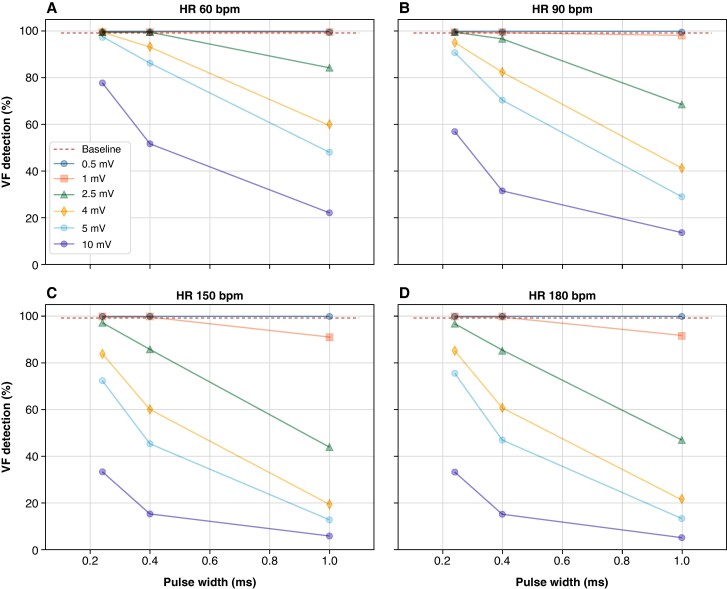
Percentage of VF episodes detected by ICD sensing and detection algorithm during simultaneous pacing at different pulse widths (*x*-axis) and voltages (see legend) for pacing rates of 60 ppm (panel *A*), 90 ppm (panel *B*), 150 ppm (panel *C*), and 180 ppm (panel *D*). The dashed line indicates the VF detection performance (99.00%) at baseline (i.e. without simultaneous pacing).

*Figure 8* presents the results of the *in-silico* computer simulation studies, showing the percentage of VF episodes detected by the ICD sensing and detection algorithm during PPG pacing conditions at different pulse widths (*x*-axis) and voltages (see legend) for pacing rates of 60 ppm (panel A), 90 ppm (panel B), 150 ppm (panel C), and 180 ppm (panel D). The red dashed line indicates the VF detection performance (99.00%) at baseline in the absence of pacing. As observed, there is good VF detection performance at low-amplitude pacing pulses and low pacing rates. The percentage of VF detection across various combinations of pacing parameters indicates that pacing pulse width has a significant impact on VF detection performance (*Figure 8*). Additionally, VF detection performance appears to be independent of the pacing rate for amplitudes of 1 mV or less and pulse widths of up to 0.24 ms.

**Figure 9:**
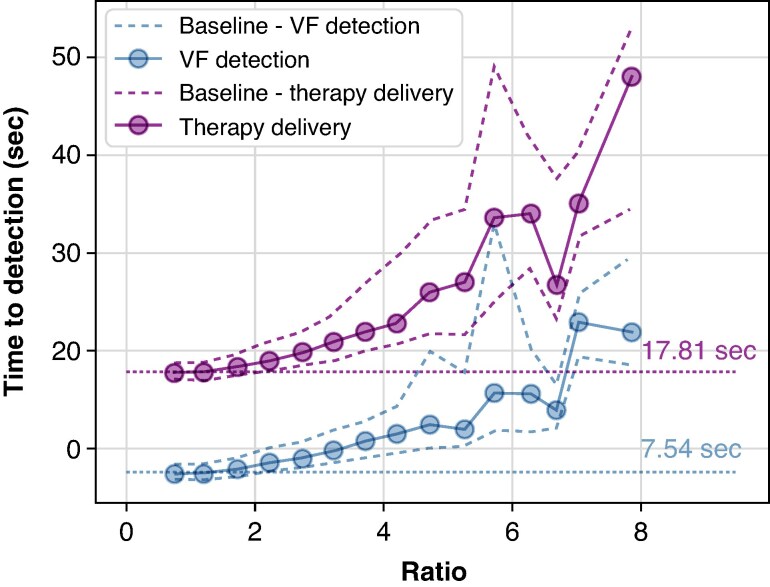
Mean time to VF detection and therapy delivery are plotted against the ratio between the mean pacing pulse amplitudes to VF amplitudes. Dashed lines show the interquartile range, while dotted lines represent the baseline performance in the absence of pacing.

**Figure 10:**
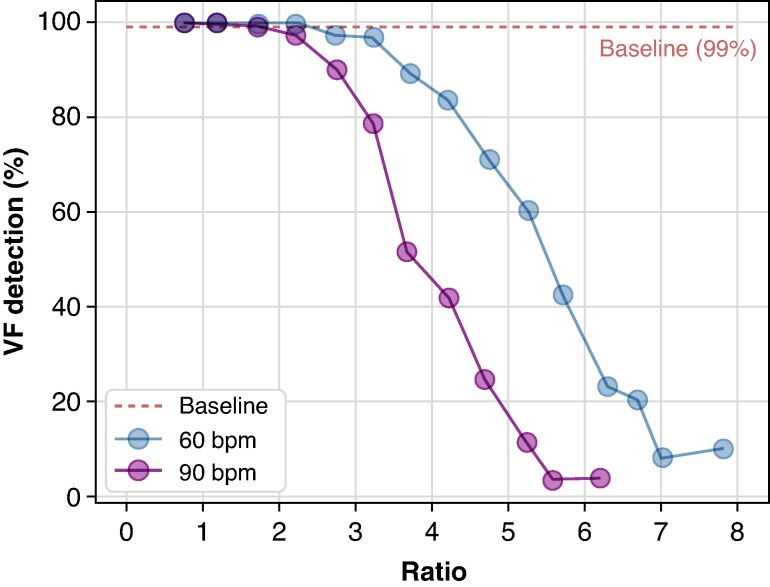
VF detection performance for pacing rates of 60 and 90 bpm as a function of the ratio between the mean amplitudes of the pacing pulses and the VF. The dashed line indicates baseline VF detection performance (99%).

*Figure 9* shows the time to detection of VF (blue) and therapy delivery (purple) vs. the ratio of pacing pulse to VF amplitude. As the amplitude of the observed pacing spike increases relative to the mean VF amplitude (bigger ratio values), the mean time to detection and therapy delivery increases. Appropriate arrhythmic detection performance decreases significantly as the pacing spike amplitude eclipses the mean VF amplitude (*Figure 10*). At 60 bpm, simulated pacing rate (a typical lower rate limit), sensed pacing spikes four times larger than the mean VF amplitude results in 80% of VF episodes being detected, while a pacing spike seven times larger than the mean VF amplitude leads to <10% detection. VF detection performance was even lower when the pacing was increased to 90 bpm.

### Saline tank studies

#### Normal conditions

*Table 1* shows the amplitude of pacing spikes sensed by the ICD for 12 combinations of PPG programmed amplitude and pulse width, evaluated at three device locations selected from our cohort of nine patients in our concomitant dataset. These locations correspond to the three patients with the shortest distance between PPG and the ICD lead. For each position, the distance from Micra tip to R2 and Micra ring to R1 ICD lead is indicated between parentheses in *Table 1*. As observed, in positions A and B, for a PPG pacing output of 3V@0.24 ms, the sensed pacing pulses are below the default programmed sensitivity of the ICD system (0.15 mV) and would not affect VF detection. For position C (shortest distance), the pacing pulses in the sensed EGM are ∼0.54 mV in amplitude, above the default programmed sensitivity of the ICD. Despite being sensed, these pacing pulses are unlikely to affect VF detection since, as demonstrated in the *in-silico* simulations, the sensed spikes are relatively small and similar in amplitude to VF. The ratio of the sensed pacing pulses to the amplitude of VF determines the risk of VF undersensing, with higher amplitudes increasing the chance for undersensing VF signals.

**Table 1:** Sensing threshold and sensed EGM amplitudes in the ICD associated with pacing pulses delivered with varying amplitudes and pulse widths and for positions A, B, and C. For each position, the distance from Micra tip to R2 and R1 ICD lead electrodes, is indicated between parentheses

PPG pacing output		ICD sensing position A (24 mm, 41 mm)		ICD sensing position B (54 mm, 20 mm)		ICD sensing position C (35 mm, 35 mm)	
Amplitude (V)	Pulse width (ms)	Sense EGM (mV)	Sensing threshold (mV)	Sense EGM (mV)	Sensing threshold (mV)	Sense EGM (mV)	Sensing threshold (mV)
1.0	0.24	0.06	0.08	0.04	0.08	0.16	0.09
1.0	0.40	0.07	0.08	0.04	0.08	0.25	0.14
1.0	1.00	0.16	0.09	0.06	0.08	0.56	0.30
1.5	0.24	0.08	0.08	0.03	0.08	0.25	0.13
1.5	0.40	0.10	0.08	0.05	0.08	0.04	0.20
1.5	1.00	0.23	0.13	0.09	0.08	1.21	0.66
3.0	0.24	0.13	0.08	0.05	0.08	0.54	0.31
3.0	0.40	0.19	0.11	0.08	0.08	1.23	0.65
3.0	1.00	0.45	0.25	0.16	0.09	2.80	1.58
5.0	0.24	0.20	0.12	0.08	0.08	1.20	0.66
5.0	0.40	0.33	0.18	0.11	0.08	1.88	0.94
5.0	1.00	1.02	0.66	0.25	0.14	4.62	2.36

**Figure 11:**
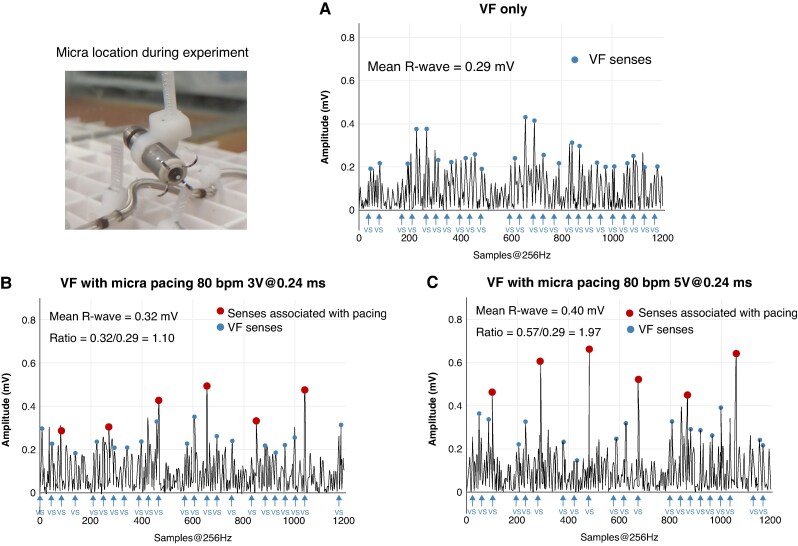
Sense EGM signal for fine VF and PPG pacing with different pacing outputs at 80 ppm. Panel *A* shows the VF amplitude sensed by the device for VF without pacing spikes alone in the absence of VF. Panel *B* illustrates the same VF signal as in panel *A* but with Micra pacing at 80 bpm using 3V@0.24 ms output. Panel *C* depicts the same as panel *B* but for pacing outputs of 5V@0.24 ms. For each case, it also shows the ratio between sensed pacing pulses and VF amplitude.

*Figure 11* shows the filtered rectified EGM signals and sense markers for the case of low amplitude VF with asynchronous pacing at position C (shortest distance in *Table 1*). Panel A displays the case of VF without pacing, while panels B and C illustrate VF with pacing at 80 bpm using outputs of 3V@0.24 ms and 5V@0.24 ms, respectively. The VF amplitude in this experiment was ∼0.29 mV, which is below the 0.86 mV mean VF amplitude from episodes detected by the EV-ICD during the EV-ICD Pivotal Trial. As observed, despite the presence of pacing pulses, the ICD reliably detected VF in all cases.

#### Closest case scenario

To further test the ICD sensing performance under pacing conditions in close proximity, a ‘closest-case’ scenario, representing an anatomically unrealistic configuration, was created in the tank. The PPG was located in close proximity to the ICD lead, as shown in *Figure 6*.

*Table 2* summarizes the programmed PPG amplitudes, the ICD sensed amplitudes, and the VF detection outcome across 12 combinations of pacing amplitude and pulse width. In this setup, a VF signal with an amplitude of 0.86 mV was injected. As shown in *Table 2*, VF was successfully detected for all programmed pacing amplitudes at 0.24 ms.

**Table 2:** Table with results on VF detection for different PPG pacing outputs injected during a VF episode of amplitude of 0.86 mV. The column ‘Amplitude’ shows the programmed pacing amplitude, ‘Pulse width’ the programmed pulse width, ‘Sense EGM’ the pacing pulse amplitude as measured by the ICD sense amplifier, the ratio between pacing spike amplitude and VF amplitude, and ‘VF detect’ whether VF was detected

Amplitude (V)	Pulse width (ms)	Sense EGM (mV)	Ratio	VF detect
5.0	1.00	6.04	7.02	No
5.0	0.40	3.75	4.36	No
5.0	0.24	2.26	2.63	Yes
3.0	1.00	5.46	6.35	No
3.0	0.40	2.13	2.48	Yes
3.0	0.24	1.39	1.62	Yes
2.0	1.00	3.67	4.27	No
2.0	0.40	1.71	1.99	Yes
2.0	0.24	1.09	1.27	Yes
1.5	1.00	2.9	3.37	No
1.5	0.40	1.26	1.47	Yes
1.5	0.24	0.82	0.95	Yes

#### Effect of pacemaker orientation

To better understand how the position of the PPG affects ICD sensing, we explored several configurations in the saline tank by rotating the PPG to four different angles (0, 45, 90, and 180 degrees), while keeping the ICD lead fixed, as shown in *Figure 7*.

**Figure 12:**
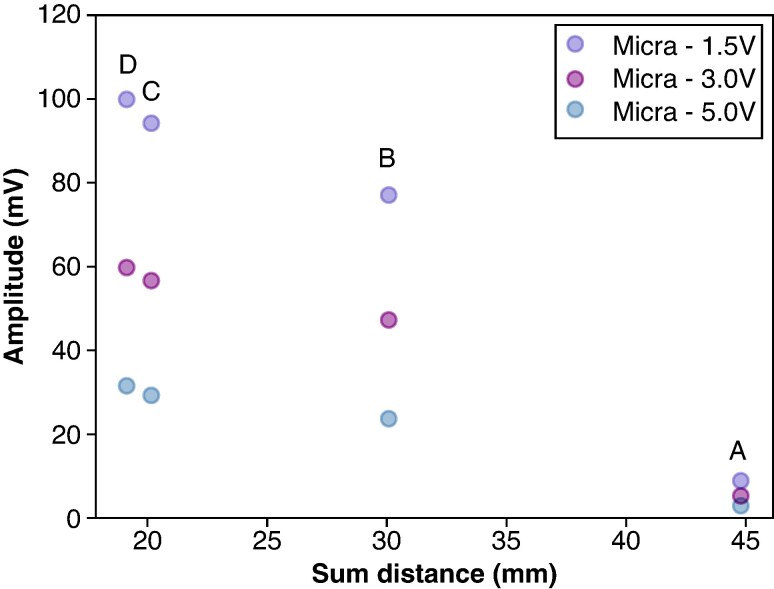
Pacing spike amplitudes vs. sum of distances of columns ‘Tip-R2 (mm)’ and ‘Tail-R1 (mm)’ in *Table 4* for locations A, B, C, and D.

To examine the effect of these changes, we plotted the voltage sensed in the ICD lead against the distance between the PPG electrodes and the ICD sensing electrodes (*Figure 12*). Interestingly, the results reveal that it is not the rotation angle itself that drives the sensing amplitude, but the distance created by the rotation. The closer the PPG electrodes are to the ICD sensing electrodes, the higher the sensed amplitude.

#### Ring1-Ring2 vs. Ring-can sensing vectors

*Table 3* summarizes the sensed amplitudes for the three configurations and the corresponding statistical analysis using the Wilcoxon signed-rank test. The Ring1-Ring2 vector exhibited significantly higher amplitudes compared with the Ring1-Can (*P* = 0.0057) and Ring2-Can (*P* = 0.0001) vectors. In contrast, the difference between the two Ring-Can configurations was not statistically significant (*P* = 0.4803).

**Table 3:** Sensed amplitudes recorded by the EV-ICD across three configurations (Ring1-Ring2, Ring1-Can, and Ring2-Can). Values are presented as median (interquartile range). Pairwise comparisons were performed using the Wilcoxon signed-rank test, with *P*-values indicating statistical significance between vectors

Vector	Amplitude (mV)	*P*-value (vs. Ring1-Ring2)	*P*-value (vs. Ring1-Can)	*P*-value (vs. Ring2-Can)
Ring1-Ring2	0.42 (0.10–1.58)	–	0.0057	0.0001
Ring1-Can	0.30 (0.10–0.99)	0.0057	–	0.4803
Ring2-Can	0.20 (0.00–0.70)	0.0001	0.4803	–

**Table 4:** Distances from PPG Tip to Ring2 (Tip-R2 column) and PPG Ring to Ring1 ICD electrode (Ring-R1 column) as result of PPG rotation for the locations depicted in *Figure 7*

Location	Rotation angle (degrees)	Tip-R2 (mm)	Ring-R1 (mm)	Tip-R2 + Ring-R1 (mm)
A	90	18.17	26.64	44.81
B	0	11.92	7.23	19.15
C	180	7.36	12.79	20.15
D	45	13.48	16.58	30.06

### Monte Carlo simulations

**Figure 13:**
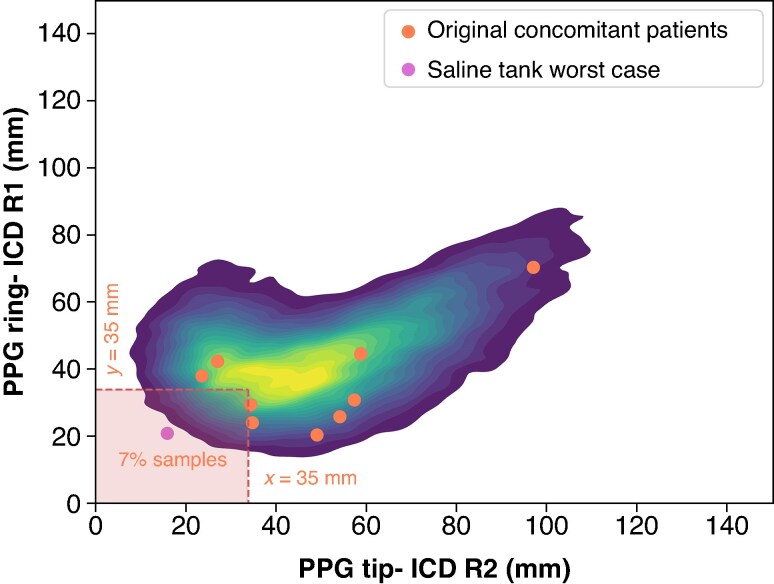
Plot with Tip-Ring2 and Ring-Ring1 distances for the nine original patients (orange dots), and an Monte Carlo simulation generating 50 000 samples (contour plot). The orchid colour dot indicates de location associated with the closest distance experiment detailed in *Figure 6*. The shaded box in the lower left contains those patients who have distances below the 35 mm distance limit.

*Figure 13* shows the outcome of a Monte Carlo simulation consisting of 50 000 samples, modelling the placement of a PPG relative to the ICD based on implant distances and probabilities observed in our cohort of nine patients. In *Figure 13*, orange points indicate the nine patient-based simulated concomitant implants, while the red point represents the closest-case configuration tested in the saline tank experiments (*Figure 6*). According to the simulation, there is a 7% probability that both Tip-Ring2 and Ring-Ring1 will be <35 mm.

### Leadless pacemaker use conditions

**Figure 14:**
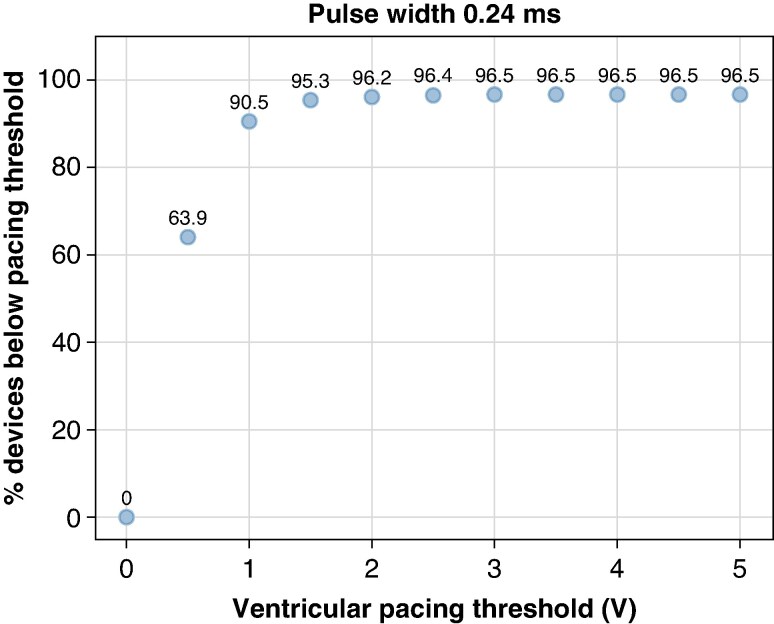
Percentage of PPG devices for different ventricular pacing thresholds and 0.24 ms pulse width observed in the DWAS dataset.

*Figure 14* shows the ventricular pacing amplitude settings for all PPGs in the DWAS database that are programmed with pulse widths of 0.24 ms or below. As supported by the *in-silico* findings above, a pulse width of 0.24 ms is key to avoid VF undersensing. This figure shows the cumulative percentage of devices (*y*-axis) programmed at or below the indicated pacing amplitude (*x*-axis).

Based on the saline tank simulations, pacing outputs of 3V@0.24 ms or lower are expected to provide near 100% VF detection performance. Even in the closest-case configuration, an anatomically unrealistic scenario, VF was reliably detected at the mean VF amplitude from the EV-Pivotal study (*Table 2*). As shown in *Figure 14*, 96.5% of devices in the DWAS database are programmed at this pacing output or lower.

To provide an additional safety margin against VF undersensing, a more conservative threshold of 1V@0.24 ms could be considered. In this setting, 90.5% of recorded PPG devices are already programmed at or below this output. Therefore, with the three times safety margin, we could safely say that nearly 90% of all PPG devices should ensure robust VF detection if paired with an EV-ICD for all device distances.

## Discussion

Traditional transvenous pacing systems have provided reliable therapy for decades, but are associated with complications such as lead failure, infection, and venous obstruction. To mitigate these limitations, less invasive solutions such as leadless pacemakers, which eliminate the need for transvenous leads and subcutaneous pockets, were developed. As reviewed by Defaye *et al.*^7^ the field has undergone substantial transformation, with leadless systems representing a paradigm shift in device design and implantation strategy. Long-term outcome data, including real-world analyses, demonstrate favourable safety and efficacy profiles of leadless pacing compared with traditional transvenous systems.^7^ In particular, 2-year results in high-risk Medicare subgroups (patients with malignancy, diabetes, COPD, tricuspid valve disease) show lower device-related complications and reinterventions with leadless single-chamber pacing and no difference in adjusted mortality, reinforcing the clinical value of minimizing transvenous hardware.^8^

Building on this technological evolution, our study provides new insights into the concomitant use of leadless pacing (Micra) with extravascular ICD (EV-ICD) technologies, both designed to avoid complications inherent to transvenous leads. Although each device individually offers advantages, their combined use has not been well characterized, raising concerns about potential issues such as oversensing pacemaker pulses by the ICD or undersensing of ventricular fibrillation due to the presence of large pacing artefacts. However, our *in-silico* simulation and saline tank experiments above provide evidence suggesting a minimal risk of over- or undersensing.

While a single device capable of both pacing and high-voltage therapy may generally be considered a safer option, this study focuses on evaluating the feasibility and interactions between a leadless pacemaker and a substernal ICD. This approach reflects real-world scenarios where patients may require both devices. For example, this configuration could be relevant for patients who already have a Micra leadless pacemaker and later develop a need for ICD therapy, or for those with an EV-ICD who subsequently require pacing support from a Micra device. Furthermore, this dual-device solution addresses clinical situations where patients are unable to tolerate traditional transvenous leads due to vascular system limitations or other contraindications, offering a viable alternative for managing complex cardiac conditions.

The *in-silico* experiments were designed to simulate EGMs recorded by the ICD under pacing conditions, derived from real VF recordings and clinical conditions, without accounting for device location and anatomical variability. The results show that a major factor determining the performance of accurate tachyarrhythmia detection is the pacing amplitude to VF amplitude ratio. The results also show that low pulse widths of 0.24 ms or less would consistently allow detection of VF (*Figure 8*). At higher pulse widths and amplitudes, there could be concerns for delayed or underdetection, particularly with higher pacing rates, which would not allow time for the dynamic sensor to detect fine voltage.

Our saline tank experiments provide further direct empirical measurement of pacing pulse amplitudes and widths in physically simulated clinical conditions. The results show that for all the tested configurations from nine simulated concomitant implants, VF was not undersensed for pulse widths of 0.24 ms or below, independently of the pacing output. This was true even for small amplitude VF (∼0.30 mV sensed EGM amplitude). Further analysis using what we considered the closest and worst scenario with Micra located as shown in *Figure 6*, showed that for a pulse width of 0.24 or less VF was also detected independently of Micra’s programmed pacing output for the mean VF amplitude of 0.86 mV (mean VF amplitude from EV-Pivotal Study). As pulse width was increased above 0.24 ms, VF undersensing was observed under different pacing outputs. These findings highlight the critical role of pulse width, more than amplitude, in preserving ICD sensing performance during concomitant pacing. Importantly, maintaining a pulse width of 0.24 ms or less not only optimizes VF detection but also aligns with clinical programming practices that prolong Micra’s battery life. Taken together, the data support 0.24 ms as a key threshold for safe and effective pacing in patients who may receive both a Micra PPG and an ICD.

The saline tank experiments were conducted using Ring1-Ring2 as the primary sensing vector. However, in clinical practice, Ring-Can configurations may be selected when low R-wave amplitudes (<1 mV) are observed at the Ring1-Ring2 vector, in order to reduce the risk of inappropriate therapy from oversensing when the sensing algorithm approaches its sensitivity threshold. The extended analysis to evaluate how pacing spike amplitudes sensed on Ring1-Ring2 translate to the Ring-Can configurations showed that the Ring1-Ring2 sensing vector consistently yields higher sensed amplitudes compared with the Ring-Can configurations. These findings imply that results obtained with the Ring1-Ring2 configuration regarding VF undersensing can be extrapolated to the Ring-Can vectors. Since the Ring1-Ring2 vector exhibits higher sensed amplitudes, the absence of VF undersensing in this vector suggests that VF undersensing is unlikely in the Ring-Can configurations with lower sensed amplitudes, and therefore less susceptibility to interference with VF detection.

In addition, in clinical scenarios where small R-wave amplitudes are observed on the Ring1-Ring2 vector (for example, 0.6–1.4 mV), clinicians may consider selecting a Ring-Can sensing configuration and programming the EV-ICD NOISE rejection setting to HIGH to reduce myopotential oversensing. It is also plausible that a higher NOISE rejection setting could attenuate pacing artefacts from a concomitant Micra device. While this approach may serve as a practical workaround in select patients, our experimental platform did not permit a rigorous assessment of this strategy: the saline setup cannot reproduce physiologic myopotential noise, and we were unable to synchronize Micra pacing with the recorded EGMs injected into the tank. As a result, the effect of higher NOISE rejection settings on Micra/EV-ICD interactions—both in terms of suppressing pacing spikes and preserving VF sensitivity—remains untested in our study. This question warrants dedicated evaluation in animal models or human clinical studies under physiologic conditions.

Additionally, the effect of the orientation angle of the Micra with respect to the ICD lead was studied during these saline tank experiments. We found that the angle was not as important as the distance between the PPG and ICD electrodes. Rotating the Micra induces changes in distance, and these changes are the key factor affecting the pacing spike amplitudes sensed by the ICD. As observed in *Figure 12*, the primary factor influencing the pacing spike amplitudes sensed by the ICD is the distance between the electrodes of both devices rather than the angle of Micra relative to the lead. This finding has important practical implications. By identifying distance as the dominant factor, we effectively reduce the degrees of freedom that must be considered during device placement assessments. This simplification may facilitate more efficient and reliable evaluation of VF sensing integrity in patients with concomitant implants.

During the saline tank experiments, only nine simulated concomitant locations were tested, which limits the extrapolation of the results to the general concomitant population. In the sample, all patients had distances between Micra and ICD electrodes of <35 mm. Furthermore, the probability of patients having inter-device electrode distances closer than 35 mm was unknown. To address this limitation, a Monte Carlo Simulation was conducted, revealing a probability of 7% for patients with distances of <35 mm. It is worth noting that this probability is overestimated, as distances <10 mm between both ICD and Micra electrodes are not possible. The reason is that bringing the PPG tip closer than 10 mm to the ICD Ring2 inherently increases the distance between the PPG ring and the ICD Ring1, making such dual proximity anatomically unfeasible. For example, in the configuration shown in *Figure 6*, the distance between the PPG tip and ICD Ring2 is 16 mm, while the distance from the PPG ring to ICD Ring1 is 21 mm. If the PPG tip were moved closer, from 16 to 10 mm, the corresponding distance from the PPG ring to ICD Ring1 would increase to 27 mm. This geometric trade-off highlights the physical constraints that limit dual proximity below 10 mm.

However, the estimated probability alone is insufficient since the risk of VF undersensing for small distances also depends on Micra’s programmed output and VF amplitude. For example, in the closest configuration studied in this research (*Figure 6*), VF was detected for the average VF amplitude of 0.86 mV (from the EV-Pivotal study) with Micra programming output of 5V@0.24 ms or less. To provide a more conservative assessment and account for patients who may have lower VF amplitudes or even closer distances (not physiologically possible), we applied a safety margin. Based on this rationale, we hypothesized that a Micra output of 1V@0.24 ms or less would ensure safe and reliable VF detection under all plausible clinical conditions.

To determine how often Micra devices are programmed within this safe range, we analysed data from the Medtronic DWAS database. The results show that indeed 90.5% of the Micra devices are programmed at1V@0.24 ms or below (*Figure 14*). Therefore, the joint probability of a Micra patient requiring pacing outputs >1V@0.24 ms [*P*(A) = 0.1] and having an electrode-to-electrode distance below 35 mm [*P*(B) = 0.07] is *P*[A&B] = 0.007 (∼0.7%) of both conditions occurring simultaneously. This suggests that between 99.3% and 100% of the patients with concomitant implants are expected to be free from the risk of VF undersensing. Given the limited data (Monte Carlo simulations based on nine virtual patients) and human variability, a more conservative estimate of successful VF sensing in patients with concomitant implants would be to apply a five-fold increased threshold (3.5%), having a free risk of VF undersensing between 96.5% and 100%.

Other methods of device-to-device communication are possible to allow the extravascular ICD to respond to messages or readings sent by the leadless device wirelessly with near field communication, or conversely have the leadless pacemaker directly working with the ICD logic similar to a standard implantable cardioverter-defibrillator. However, there are several logistical and practical hurdles with such a setup. Messages would have to be authenticated to ensure originating from the known/paired device and received accurately. Graceful handling of breaks in telemetry would require logic for the two devices to function independently, negating a great deal of potential benefits. Constant telemetry would require increased power draw from both devices, and on-demand would still require always-on listening circuitry. Additionally, discordant readings between the implantable pacemaker and the ICD would have to be adjudicated, and troubleshooting would be complex. A pacemaker with dynamic sensing circuitry on board to completely obviate sensing from the ICD would require a pacemaker re-design and not have utility in the majority of pacemaker-only use cases.

Finally, while our modelling and saline tank experiments provide *in-silico* and *in-vitro* evidence supporting the safe coexistence of Micra and EV-ICD systems under clinically realistic pacing conditions, these findings are now beginning to be echoed in real-world clinical practice. Two recently published case reports have documented the first successful simultaneous use of Micra and EV-ICD systems in patients with no other viable implant options. In the case presented by Sterliński *et al.*,^9^ both devices were implanted in a 19-year-old with fulminant post-infectious cardiomyopathy, demonstrating safe and effective performance with customized programming over a 3-month follow-up. Similarly, Moltrasio *et al.*^10^ described the first-in-human open surgical implantation of an EV-ICD with the lead sutured directly to the right ventricular wall, alongside a Micra AV, in a patient with active endocarditis and prior device infections. These early reports support the clinical viability of this leadless and extravascular combination.

Additional support for this paradigm comes from a broader body of literature evaluating the combination of Micra with subcutaneous ICDs (S-ICDs). Case series and reports^11–14^ have demonstrated that the Micra + S-ICD combination can function safely without oversensing, double counting, or interference, even in high-risk patients and during defibrillation testing.

Taken together, these clinical experiences and our experimental results support the conclusion that leadless pacemakers and extravascular ICDs can coexist safely under appropriate conditions. By adhering to programming strategies such as limiting pacing pulse width to ≤0.24 ms and accounting for inter-electrode distance, the majority of concomitant implant patients could be protected from VF undersensing. This strengthens the case for expanding access to fully leadless ICD-pacing combinations in patients for whom transvenous options are undesirable or contraindicated.

### Study limitations

This study combines *in-silico* simulations with an *in-vitro* saline tank platform. The tank does not reproduce anisotropic thoracic conduction, tissue interfaces, respiration, and posture-related variability, and other physiologic interactions.

Furthermore, the generalizability of these results is limited to the devices evaluated (Micra leadless pacemaker model and one EV-ICD) and should not be extended to other devices or manufacturers without dedicated testing. In addition, although interference was not observed under our tested conditions, the risk cannot be considered negligible, and the potential clinical consequences could be significant.

Another limitation stems from the sensing vector used in our experiments (Ring1-Ring2). Measurements were performed primarily using the Ring1-Ring2 sensing vector with extrapolation to Ring-Can where we observed less impact of pacing spikes sensed by the EV-ICD. In fact, we did not systematically assess the effect of all sensing parameters (e.g. NOISE rejection setting as HIGH to avoid myopotential detection in Ring-Can configurations), and therefore cannot determine their impact from this work.

The pacing scope was limited to leadless VVIR; we did not assess conduction system pacing (e.g. LBBAP) or biventricular CRT-P, which differ in timing, output, and VF detection profiles, and results should not be extrapolated to those modalities without targeted evaluation. In addition, we did not evaluate scenarios involving more than one leadless pacemaker (e.g. combination of atrial and ventricular leadless pacemakers). An atrial device may sit at shorter distances to the EV-ICD sensing vector, potentially increasing sensed pacing artefact amplitudes. This could raise the risk of oversensing and double-counting.

Finally, we did not evaluate specific programming strategies (e.g. Acute Phase settings, sensitivity adjustments, NOISE rejection). Any consideration of such strategies should be individualized and guided by clinician judgment, with verification through in-clinic testing and EGM review; formal device labelling and guidance for concomitant use are not yet established.

## Conclusions

Computer modelling and saline tank experiments suggest that concomitant use of an EV-ICD with a leadless pacemaker may be feasible under the tested *in-silico* and *in-vitro* conditions designed to approximate clinical scenarios. In our models, the likelihood of VF undersensing or inappropriate sensing of pacemaker pulses was low. The risk of VF undersensing increases with high pacing outputs, particularly with extended pulse width, and close proximity to the ICD lead (<35 mm). These conclusions are limited to the specific device models evaluated and should not be generalized to other devices or manufacturers without dedicated testing. Given the *in-vitro* nature of this research, further clinical investigations are necessary. Although the likelihood of VF undersensing was low in our results, the risk cannot be considered negligible, and its potential clinical consequences are significant. Therefore, close observation of concomitant human implants as they occur should take place to aid in the confirmation and generalization of these results.

## Data Availability

The data underlying this article cannot be shared publicly due to confidentiality agreements with the device manufacturer. The data will be made available to qualified researchers upon reasonable request from the corresponding author.
